# Editorial: RNA Regulation in Development and Disease

**DOI:** 10.3389/fgene.2020.00430

**Published:** 2020-04-28

**Authors:** Pascal Chartrand, Maritza Jaramillo, Chiara Gamberi

**Affiliations:** ^1^Department of Biochemistry and Molecular Medicine, Université de Montréal, Montréal, QC, Canada; ^2^Institut National de la Recherche Scientifique (INRS) – Centre Armand-Frappier Santé Biotechnologie, Laval, QC, Canada; ^3^Biology Department, Concordia University, Montreal, QC, Canada

**Keywords:** RNA, translational control, RNA-binding proteins, mRNA localization, development, disease

A wide variety of post-transcriptional regulatory events in the life of an mRNA have emerged as major checkpoints during its temporal and spatial journey within the cell. The advent of deep sequencing technologies combined with various fractionation or enrichment protocols has produced a wealth of data regarding transcripts, their variants and their interactomes. Yet, these data must be integrated with mechanistic and biological frameworks in order to better understand complex and dynamic regulatory networks that tailor mRNA metabolism and shape the cell proteome in healthy and diseased states.

The articles in this Research Topic review our current knowledge in eukaryotic post-transcriptional gene regulation, from mRNA export out of the nucleus to its localization, translation, and eventual decay. Among several topics related to RNA regulation, this article collection puts a particularly strong emphasis on translational control (i.e., regulation of mRNA translation efficiency) and its impact on localized protein synthesis, downstream transcriptional programs, cellular metabolism, organismal development, and disease pathogenesis.

First, several articles review fundamental RNA-based mechanisms of post-transcriptional gene regulation. Starting in the nucleus, Palazzo and Lee describe the various cis-acting determinants regulating nuclear retention or export of both long non-coding and coding RNAs. Once in the cytoplasm, mRNAs can be sorted to specific subcellular domains, allowing localized translation of these transcripts. In their article, Neriec and Percipalle present the different mechanisms behind this process, focusing on CBF-A/hnRNP AB-mediated mRNA transport and localization. The role of the 3'UTR in modulating mRNA localization, but also its translation and fate, are reviewed by Mayya and Duchaine. Finally, Karamyshev and Karamysheva discuss various mechanisms involved in quality control of both mRNAs and proteins during translation to prevent production of abnormal proteins.

A second group of articles in this collection focuses more specifically on the roles of RNA-mediated control of cellular metabolism and organismal development. Necessary to produce biological building blocks, regulated translation is key for cell growth and is a downstream target of several signaling pathways that control cellular metabolism. One example is the mammalian or mechanistic target of rapamycin (mTOR) signaling pathway. A review by Cao discusses novel rhythmic functions of mTOR signaling in translational control in neurons, as they regulate their metabolism to suit circadian functions. Another example is the role of ribosome availability in regulating cellular metabolism and the cell cycle. While ribosomes have been considered for a long time as mere executants in the translation program, Calamita et al. discuss novel evidence of ribosome heterogeneity and its impact on differential mRNA translation and ribosomopathies, diseases in which these processes malfunction.

Translational control also plays important developmental functions such as stem cell differentiation, which is the topic of a review by Tahmasebi et al., who describe several mechanisms that control mRNA translation to coordinate stem cell renewal and differentiation. Particularly important during early development, mRNA localization has been extensively researched in *Drosophila*. While original studies were carried out in the oocyte and early embryo, most mRNA localization factors are conserved evolutionarily and are expressed in multiple tissues at late developmental stages and the adult, suggesting that RNA localization may be necessary throughout the lifespan of many organisms to enable structural and functional cellular asymmetries. Hughes and Simmonds illustrate the diversity of mRNA localization patterns in *Drosophila*, its role of sorting proteins to various subcellular compartments and reflect on the conservation of the underlying regulation. Finally, this section also includes two original research articles, one on the global transcriptome of adipogenic differentiation in cattle by Cai et al., and a second article by Alard et al. on the regulation of the translation initiation factors eIF4Gs by the proteasome.

The third section of this collection includes several articles on the roles of RNA regulation and mis-regulation in diseases. There is growing appreciation that sets of mRNAs encoding functionally related proteins are coordinately regulated through Untranslated Sequence Elements for Regulation (USER) codes that are “read” by specific RNA-binding proteins. This post-transcriptional regulatory mechanism, referred to as the RNA regulon model, is reviewed by Culjkovic-Kraljacic and Borden, who discuss the concepts of one- and two-tier RNA regulons and explain how their mis-regulation is a feature of diseases such as cancer. Moreover, the authors highlight how the advent and integration of “OMICS” approaches (e.g., RIP-seq, CLIP-seq, RIP-ChIP, etc.) has contributed to uncover the RNA-interactome of RNA-binding proteins and the therapeutic potential of redirecting RNA regulons. Dysregulation of signaling cascades or mis-expression of translation initiation factors frequently occurs in cancers, which impact translation initiation (a key, highly regulated step) and cell growth. This topic is reviewed by Hernández et al., with a focus on the development of pharmacological inhibitors of translation initiation as a potential treatment for prostate cancer. Translational output can also be affected by mutations in the sequence of a transcript, and a review article by Robert and Pelletier discusses how single nucleotide polymorphisms (SNPs) in regulatory elements of an mRNA (5′UTR, 3′UTR, uORF, miRNA-binding site, etc.) can impact its translation.

RNA molecules are also at the forefront of human action against infectious diseases. Efficient innate immune responses to bacterial, protozoan, fungal, and viral pathogens are largely dependent on a delicate balance between transcriptional and post-transcriptional regulation of genes encoding pro- and anti-inflammatory mediators. Ostareck and Ostareck-Lederer describe in their review key RNA-binding proteins (i.e., HuR, TTP, hnRNP K, and TIA-1/TIAR) that coordinate macrophage inflammatory responses to the Gram-negative bacterial endotoxin lipopolysaccharide by controlling turnover and translation of immune-related transcripts. Non-coding RNAs from bacteria such as ribozymes, riboswitches, and CRISPR-Cas9 systems are being developed as potential antimicrobials to curb acquired multi-drug resistance in pathogens without harming beneficial microbiota, as reviewed by Di Noto et al.. Finally, the current COVID-19 pandemic reminds us that RNA viruses, such as coronavirus and flavivirus, remain among the most formidable challenges faced by today's world health system. Flavivirus (such as dengue, Zika, West Nile, and yellow fever viruses) and the fates of the flavivirus RNA genome are the topic of a review by Mazeaud et al..

The last two review articles focus on RNA regulation in the nervous system, where eIF4E-dependent translational control plays a major role in regulating the brain response to pain and the development of chronic pain diseases (Uttam et al.). Last but not least, RNA dysregulation is a key contributor in several neurodegenerative disorders, such as amyotrophic lateral sclerosis (ALS), frontotemporal degeneration (FTD), and microsatellite expansion disorders such as Fragile X syndrome. A review by Butti and Patten describes how major genes mutated in ALS, such as *SOD1, TARDBP, FUS*, and *C9orf72*, are all involved in various aspects of RNA metabolism.

As the field of RNA Biology advances very rapidly and the integrative analysis of global-scale RNA-protein interactions continues to evolve, novel examples of sophisticated regulatory mechanisms of RNA metabolism will emerge and thereby improve fundamental knowledge of cellular and organismal physiology. We may thus begin to comprehend how widespread, yet selective changes in transcriptional and translational programs underscore normal biological rhythms and adaptations to the changing environment. A better understanding of the role of dysregulated RNA metabolism in disease pathogenesis will be instrumental to design targeted RNA-based therapeutics to combat morbidity and mortality related to pathological conditions that affect millions of people around the world.

## Author Contributions

All authors listed have made a substantial, direct and intellectual contribution to the work, and approved it for publication.

## Conflict of Interest

The authors declare that the research was conducted in the absence of any commercial or financial relationships that could be construed as a potential conflict of interest.

